# X-linked disorders with cerebellar dysgenesis

**DOI:** 10.1186/1750-1172-6-24

**Published:** 2011-05-15

**Authors:** Ginevra Zanni, Enrico S Bertini

**Affiliations:** 1 Unit of Molecular Medicine, Departement of Neurosciences, Bambino Gesù ediatric Research Hospital, 4 Piazza S. Onofrio, 00165 Rome, Italy

## Abstract

X-linked disorders with cerebellar dysgenesis (XLCD) are a genetically heterogeneous and clinically variable group of disorders in which the hallmark is a cerebellar defect (hypoplasia, atrophy or dysplasia) visible on brain imaging, caused by gene mutations or genomic imbalances on the X-chromosome. The neurological features of XLCD include hypotonia, developmental delay, intellectual disability, ataxia and/or other cerebellar signs. Normal cognitive development has also been reported. Cerebellar dysgenesis may be isolated or associated with other brain malformations or multiorgan involvement. There are at least 15 genes on the X-chromosome that have been constantly or occasionally associated with a pathological cerebellar phenotype. 8 XLCD loci have been mapped and several families with X-linked inheritance have been reported. Recently, two recurrent duplication syndromes in Xq28 have been associated with cerebellar hypoplasia. Given the report of several forms of XLCD and the excess of males with ataxia, this group of conditions is probably underestimated and families of patients with neuroradiological and clinical evidence of a cerebellar disorder should be counseled for high risk of X-linked inheritance.

## Disease names and synonyms

X-linked Congenital Ataxias

X-Linked Disorders/Syndromes with Cerebellar Dysgenesis

## Definition and classification

The term X-linked disorders with cerebellar dysgenesis (XLCD) is used here to describe an emerging group of rare conditions in which the hallmark is a cerebellar defect, visible on neuroimaging, caused by gene mutations or genomic imbalances on the X-chromosome. A classification of this group of rare disorders is still difficult because in most cases the pathogenesis is unknown. The best characterized forms are X-linked syndromes with associated cerebellar hypoplasia due to *OPHN1 *or *CASK *gene mutations. In other X-linked disorders like Fragile X, Oral-facial-digital type I or Opitz GBBB syndromes, cerebellar defects are increasingly being recognized.

## Epidemiology

The overall incidence of congenital cerebellar malformations is high, 1/4,000-5,000 live births, the contribution of X-linked forms is probably underestimated.

## Clinical and molecular description

### a. X-linked non-progressive congenital ataxias

**Figure 1:**
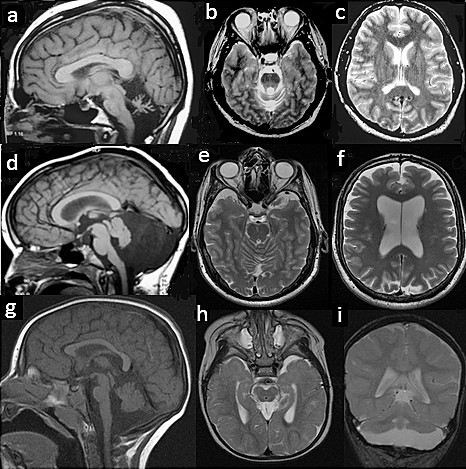
**Brain MRI studies of patients with XLCD**. 1 a-c (male proband of a family with X-linked congenital ataxia) Midsagittal T1-weighted image showing an overall size reduction of the cerebellum predominant in the vermis with widened interfolia sulci. Brainstem is normal (a). Axial T2-weighted image showing global cerebellar hypoplasia without dysplasia (b). Axial T2-weighted image showing normal cortex and ventricles (c). d-f (male patient carrying a *OPHN1 *mutation): midsagittal T1-weighted image showing hypoplastic cerebellar vermis and enlarged cisterna magna (d) axial T2-weighted image showing cerebellar hypoplasia and vermis dysplasia (e) axial T2-weighted image showing lateral ventricle dilatation and mild cortical atrophy (f). g-i (female patient carrying a *CASK mutation*): Midsagittal T1-weighted image showing moderate vermis hypoplasia and flat pons (g). Axial T2-weighted image showing simplified gyral pattern and ventricular dilatation (h). Coronal T2-weighted image showing global cerebellar hypoplasia and simplified cortical gyri (i).

Congenital ataxias (CA) were first defined by Batten in 1905 [1] as "cases in which ataxia has been noted early in life and in which there is a tendency to gradual improvement". This heterogeneous group of disorders accounts for 10% of non-progressive infantile encephalopathies [2]. The first X-linked form was described in a large family of Eastern Russian descent with seven affected males over three generations. All patients had markedly delayed early developmental milestones, cerebellar ataxia, external ophthalmoplegia, pyramidal signs, normal cognitive development and a non-progressive course. Neuroimaging studies revealed marked hypoplasia/atrophy of the cerebellar vermis and hemispheres. Linkage studies mapped the gene to a large genetic interval on Xp11.21-Xq24 with a maximum lod score of 4.66 at DXS1059 [3]. A second family was reported with a similar phenotype, the propositus and his maternal uncle manifesting severe hypotonia at birth, psychomotor delay, slow eye movements, non progressive ataxia and normal intelligence [4]. Neuroimaging studies revealed global cerebellar hypoplasia/atrophy not evident in the first years of life. Despite the cerebellar changes, the clinical course was non-progressive, as it is expected in this group of ataxias. A genetic interval, partially overlapping with the first reported family, was identified by exclusion mapping. A large family of Norwegian descent with six affected males over three generations with non-progressive X-linked congenital ataxia and normal cognitive development was reported [5]. Hypotonia and overall delay of motor development were noted since birth. Nystagmus, dysarthria and trunkal ataxia were present. Neuroimaging studies showed global hypoplasia/atrophy of the cerebellum, predominant in the vermis (Figure 1a-c) The condition was mapped to a genetic interval of 12 Mb at Xq25-q27.1 with a maximum lod score of 3.44 at DXS1192 not overlapping with the previously identified locus, indicating that there are at least two genes responsible for this rare forms of X-linked congenital ataxia.

### b. X-linked genes/syndromes with cerebellar dysgenesis

#### Oligophrenin-1 syndrome

The *OPHN1 *syndrome (MIM 300486) was first described in a 12 year-old girl, carrier of a *de novo *translocation t(X;12)(q11;q15) which was found to disrupt the *Oligophrenin-1 *(*OPHN1) *gene at Xq12 and encodes a RhoGTPase activating protein (RhoGAP) involved in synaptic morphogenesis and function [6-8]. The girl had congenital hypotonia, severe developmental delay, moderate cognitive impairment, early-onset complex partial seizures and bilateral divergent strabismus and dysmetria. At a later reevaluation, Brain MRI showed a posterior vermis dysgenesis including partial agenesis of lobules VI and VII associated with right vermian parasagittal cleft and mild cerebellar hemisphere hypoplasia. A consistent dilatation of the lateral cerebral ventricles and mild cortical atrophy was also present. A similar clinico-radiological phenotype (Figure 1a-c) was found in the affected males of a family carrying a single nucleotide deletion in exon 19 of the *OPHN1*, initially diagnosed with non-syndromic X-linked mental retardation [9]. Another deletion comprising exon 19 of the *OPHN1 *gene, was identified in a family with five affected males showing moderate to severe mental retardation, infantile-onset epilepsy, hypotonia, strabismus and ataxia. Cryptorchidism and genital hypoplasia were present in all affected males. Neuroradiological findings included cortical atrophy, enlargement of the cerebral ventricles, bilateral hypoplasia of the head of the caudate nucleus, lower vermis and cerebellar hemisphere hypoplasia. One carrier sister had mild learning disabilities and strabismus but her brain MRI was normal [10]. A stop mutation in exon 3 and an 8 base-pair insertion in exon 9 of the *OPHN1 *gene were identified in two other families with a similar phenoype [11]. In a family with a 2 base-pair deletion in exon 8 of *OPHN1 *leading to a premature stop codon, the affected males showed developmental and cognitive delay with IQ ranging from 46 to 54, strabismus, early-onset generalized tonic-clonic seizures, abnormal behaviour and a characteristic facial phenotype with long face, prominent forehead, infraorbital creases, deep set eyes, upturned philtrum and large ears. The obligate carrier females showed only mild cognitive impairment and subtle facial changes. Brain MRI studies performed in the propositus at age 3 months showed underdeveloped frontal lobes, loss of brain volume with enlarged lateral ventricles, prominent subarachnoid spaces and a particular square shape of the frontal horns. Cerebellar hypoplasia and vermis dysgenesis with a large cisterna magna and a retrocerebellar cyst were noted [12].

A *de novo *translocation disrupting the *OPHN1 *gene in a female patient and intragenic deletions of *OPHN1 *detected by arrayCGH, in a male patient and in two families in which affected individuals all showed the characteristic clinico-radiological phenotype associated with *OPHN1 *syndrome were also reported [13-16]. In one family with six affected males with intellectual deficit, minor facial anomalies, cubitus valgus, with or without sensorineural deafness, a deletion of exons 16-18 in the *OPHN1 *gene was identified [17]. Screening *OPHN1 *in a large cohort of patients led to the identification of mutations in 12% of individuals with intellectual deficit and cerebellar hypoplasia, suggesting that the screening of this gene should be implemented in boys with vermis hypoplasia, enlarged cerebral ventricles and developmental delay [18].

#### CASK syndrome

*CASK *syndrome (MIM 300749) was first described in a 4 year-old girl, carrier of an apparently *de novo *paracentric inversion 46X, inv(X)(p11.4p22.3) disrupting the Calcium/Calmodulin dependent serine protein kinase (*CASK*) gene, located at Xp11.4, which encodes a multidomain scaffolding protein that belongs to the membrane-associated guanylate kinase protein family of proteins found at neuronal synapses involved in the trafficking, targeting and signalling of ion channels [19,21]. The patient showed marked congenital and postnatal microcephaly, severe developmental delay, seizures and sensorineural hearing loss. She had minor facial anomalies: low forehead, hypertelorism, broad nasal bridge, smooth philtrum, large ears, micrognathia. She also had episodic hyperpnea and optic disc pallor with anisocoria. Brain MRI showed cerebellar hypoplasia predominant in the vermis, a small pons with flattened basis pontis, mildly enlarged 4th ventricle and reduced number and complexity of cortical gyri (Figure 1g-i). By screening a series of patients with microcephaly, developmental delay and pontine and cerebellar hypoplasia, two heterozygous deletions identified by arrayCGH and one missense mutation in exon 21 leading to a premature stop codon, were found in three girls with a clinical and neuroradiological phenotype very similar to the first described patient. A hemizygous mutation partially affecting the splice of exon 9 in a severely affected boy who died at two weeks was also found [20]. Mid-hindbrain hypoplasia was more severe, the corpus callosum was thin and unmyelinated and the cortex showed area of pachygyria. Neuropathologic examination of the cerebellum showed: poorly formed and unbranched folia, a virtually absent internal granular layer and an abnormally thick external granular layer. The molecular layer was hypercellular and disorganized. A missense mutation of *CASK *which partially disrupts the splicing of exon 2, was identified in a family with three affected males diagnosed with FG syndrome (MIM 305450) and congenital hypotonia, chronic constipation, severe mental retardation, bilateral sensorineural deafness, seizures and hyperactive, aggressive behaviour. The mother of the propositus showed mild intellectual impairment and experienced absence attacks. In addition, all mutant males had relative macrocephaly and minor facial anomalies: broad forehead, frontal upsweep of the hair, hypertelorism, saddle-root of nose, long philtrum, and micrognathia. Brain MRI of the propositus was normal [22]. Three novel missense mutations and one splice site mutation of *CASK *were found in 4 families with mild to moderate X-linked mental retardation and congenital nystagmus. Affected individuals had normal head circumference or relative macrocephaly. Brain imaging was only performed in 2 of the 4 families: in the propositus of one family MRI showed pachygyria and cerebellar hypoplasia, while in the other family with half of the affected individuals having normal cognitive development, MRI was reported normal. Ocular findings included: strabismus, cataracts, myopia or reduced visual acuity. Unsteady gait and seizures were present in some but not all affected individuals. In 2 additional families with apparently non-syndromic XLMR, novel missense mutations in exon 8 and 27 of the *CASK *gene were identified [23]. Thus the phenotypes associated with *CASK *mutations range from mild MR with or without congenital nystagmus, to severe cognitive impairment associated with cerebellar and pontine hypoplasia and abormalities of cortical development.

#### Christianson syndrome

Christianson syndrome (MIM 300243) was first described in a South African family of Norwegian descent in which 16 affected males manifested profound MR, absence speech, early-onset generalized tonic-clonic epilepsy, bilateral ophthalmoplegia and truncal/gait ataxia. Three of the 10 obligate carrier females had mental retardation. In the patients, developmental milestones were severely retarded and hypotonia was present. The chest was narrow, limb muscles were poorly developed, and joints were stiff and contractured. MRI scan showed a prominent cisterna magna, enlarged 4th ventricle, cerebellopontine and supracerebellar cisterna, hypoplasia/atrophy of the cerebellum predominantly of the vermis and pons. Most of the affected males in this family died prematurely, around age 25-30 years. Neuropathological examination showed an atrophic cerebellum, especially of the vermis. The molecular layer displayed microcystic changes and there was widespread neuronal loss in the granular and PC layers, in the pons and in the hippocampi. The condition was mapped by linkage analysis studies to Xq24-q27 [24]. In this family, a 2 base-pair deletion within the coding sequence of the *Solute Carrier family 9 member 6 *(*SLC9A6) *was identified [25]. *SLC9A6 *is located at Xq26.3 and encodes a sodium/hydrogen exchanger protein 6 (NHE6) which regulates the endoluminal pH of early endosomes involved in the trafficking of proteins essential for growth and maintainance of dendritic spines [26]. Additional mutations of *SLC9A6*: a 6 base-pair deletion, a nonsense mutation R468X, a splice site mutation causing skipping of exon 3, were identified in three other families with an Angelman-like phenotype characterized by developmental delay, postnatal microcephaly and epilepsy occurring between 9 and 26 months of age. Patients exhibited ataxia, hyperkinetic movements and frequent smiling with episodes of unprovoked laughter, open mouth, profuse drooling and swallowing difficulties, gastroesophageal reflux and thin body habitus. MRI studies performed 3 years later in one affected child showed a mild progression of the cerebellar atrophy. Proton Magnetic Resonance spectroscopy (MRS) showed elevated glutamate/glutamine in the basal ganglia of affected individuals. An additional family of 6 affected males with severe mental retardation, absent speech, autism spectrum disorder, epilepsy, late-onset ataxia, weakness and dystonia with stereotyped hand movements was reported and an in-frame 9 base-pair deletion in *SLC9A6 *was identified. Brain MRI showed moderate ventricular enlargement and sulcal widening due to atrophy of the brain, thin corpus callosum but normal cerebellum and brainstem. Post-mortem examination of two affected males showed widespread neuronal loss and deposits of the microtubule-binding protein tau in cortical and sub-cortical regions which are generally the defining neuropathological characteristics of a number of adult-onset neurodegenerative disorders but had never been seen before in early- onset cognitive delay or autism [27].

#### Fragile × syndrome

The Fragile × (FRAXA) syndrome (MIM 300624), is the most frequent cause of monogenic mental retardation with an estimated prevalence of 1/4000 males and 1/8000 females. In addition to cognitive deficits, the phenotype of the syndrome include mild facial anomalies (prominent jaw, high forehead, large ears), and macroorchidism. Many patients also manifest attention deficit hyperactivity disorder and autistic-like behavior. The syndrome is caused by a massive (more than 200) CGG triplet expansion in the 5' untranslated region of the *FMR1 *gene which results in transcriptional silencing of the gene [28]. Loss of function of the FMRP protein leads to abnormalities of dendritic spine maturation associated with altered synaptic plasticity and cerebellar motor learning (eyeblink conditioning) also seen in FRAXA patients [29]. A comparative study of the posterior fossa of a group of mutated patients and a group of age-matched controls revealed a significant reduction of the posterior vermis (lobules VI-VII) in the Fragile × population [30,31]. These data were recently confirmed by MRI morphometric studies in 3D [32].

#### Fragile X-associated Tremor/Ataxia syndrome

Fragile X-associated Tremor/Ataxia syndrome (FXTAS) is characterized by adult-onset progressive intention tremor and gait ataxia affecting more than 33% of male and 10% of female carriers of expanded CGG triplets alleles in the premutation range (50-200 repeats) of the *FMR1 *gene and is uncoupled from FRAXA. Associated features include cognitive decline, peripheral neuropathy, dysautonomia [33]. The severity of both clinical and neuropathological phenotypes (intranuclear inclusions, especially abundant in the cerebellum) is correlated with the extent of the CGG expansion, which in contrast to FRAXA, leads to a marked increase of *FMR1 *transcription. The consequence may be an RNA based "toxicity" affecting the development and long-term function of neural cells. Until recently FXTAS was defined as a late-onset neurodegenerative disorder, but evidence from animal models and from observations of children with elevated levels of *FMR1 *mRNA showing developmental delay, behaviour difficulties and increased seizure activity, suggest that the underlying pathogenic process may begin at or before birth [34]. Neuroimaging studies of affected individuals show global loss of brain volume, in particular cerebellar and cortical atrophy and hyperintensity white matter lesions around the periventricular area and in the middle cerebellar peduncles [35].

#### Hoyeraal-Hreidarsson syndrome

Hoyeraal-Hreidarsson syndrome (HHS) (MIM 300240) is a rare X-linked recessive disorder, characterized by intrauterine growth retardation, microcephaly, intellectual deficit, bone marrow failure, cancer predisposition and cerebellar hypoplasia [36]. It is caused by mutations of *DCK1 *gene located on Xq28, encoding the nucleolar protein dyskerin which interacts with the human telomerase RNA complex [37]. Detailed neuroimaging studies of an HHS patient showed global cerebellar hypoplasia, a small brainstem, thin corpus callosum and cerebral calcifications [38].

#### X-linked sideroblastic anemia with ataxia

X-linked sideroblastic anemia with ataxia (XLSA/A) (MIM 301310) is a rare X-linked disorder characterised by early onset slowly progressive ataxia associated with a sideroblastic anemia due to mitochondrial iron accumulation. It is caused by mutation of the *ABC7 *gene which maps to Xq13 and encodes an ATP-binding mitochondrial iron transporter located in the inner mitochondrial membrane, involved in mitochondrial iron homeostasis as well as in the maturation of cytosolic iron-sulfur proteins [39]. Neuroimaging studies showed global cerebellar hypoplasia with signs of atrophy in affected individuals [40].

#### Oral-Facial-Digital type I/X-linked Joubert syndrome

Oral-Facial-Digital type I (OFDI) (MIM 311200) is a rare male lethal X-linked dominant syndrome characterised by malformations of the mouth, face and digits in affected females. CNS anomalies have been found in 40% of ODFI patients, in particular vermis hypoplasia, corpus callosum agenesis, hydrocephalus and periventricular heterotopia [41]. *OFD1 *maps to Xp22 and encodes a ciliary protein. *OFD1 *mutations have been identified in two families with retinitis pigmentosa, postaxial polydactyly and the molar tooth sign characteristic of Joubert and Joubert-related syndromes [42,43].

#### X-linked Opitz/GBBB syndrome

Opitz GBBB syndromes (MIM 300000) are X-linked recessive or autosomal dominant conditions characterised by developmental delay and midline malformations: hypertelorism, cleft lip/palate oesophagolaryngotracheal defects, imperforate anus and hypospadias [44,45]. The X-linked form is caused by mutations in the *MID1 *gene which maps to Xp22.22 and encodes a microtubule-associated protein involved in ribonucleoprotein complex stabilization and ubiquitination [46,47]. Anterior vermis hypoplasia and corpus callosum abnormalities were found in more than one third of patients with *MID1 *mutations [48,49] and lack of *mid1 *in the mouse causes abnormal development of the anterior cerebellar vermis [50].

#### X-linked heterotaxy

X-linked heterotaxy (MIM 306955) is caused by mutations of the Zinc finger transcription factor *ZIC3 *located in Xq26 and encodes a transcription factor involved in the establishment of the left/right axis in early embryonic development [51]. Vermis hypoplasia is found in X-linked heterotaxy and has been reported in males hemizygous for *ZIC3 *mutations [52,53]. Cerebellar dysgenesis predominant in the flocculo-nodular lobe was described in *zic3 *deficient mice [54].

#### X-linked cortical dysgenesis

The gene *DCX *located in Xq22 and encodes the Doublecortin protein, mutated in 40% of type I lissencephaly. This cortical dysgenesis is associated with severe mental retardation and seizures in affected males and subcortical laminal heterotopia in heterozygous females [55,56]. Doublecortin is a microtubule associated protein which participates in the assembly and stabilization of microtubules in migrating neuronal cells. 25% of male patients with *DCX *mutations have mild cerebellar hypoplasia, predominant in the vermis [57,58].

The *ARX *gene located in Xp21, encodes an Aristaless-related homeobox transcription factor involved in multiple aspects of cortical development [59,60]. In a severe case of X-linked lissencephaly, absent corpus callosum and ambiguous genitalia (XLAG) due to a mutation in *ARX*, neuroimaging studies showed a hypoplastic vermis and enlarged 4th ventricle [61].

The gene *FLNA *maps to Xq28 and encodes Filamin 1, a protein interacting with F-actin, involved in brain neurogenesis and neuronal migration [62]. *FLNA *mutations cause several different syndromes with a predominant skeletal phenotype: Oto-palato digital type I and II, Melnick Needles dysplasia, frontometaphyseal dysplasia, FG-like syndrome [63]. Mutations of *FLNA *have also been found in a high proportion of individuals with bilateral periventricular nodular heterotopia, megacisterna magna, cardiovascular malformations and epilepsy. Almost all patients with *FLNA *mutations had mild to moderate cerebellar hypoplasia [64].

#### Rett syndrome

Rett syndrome (MIM312750) is an X-linked dominant neurodevelopmental disorder which represents the most important cause of severe mental retardation in the female population (1/10,000). Most cases are due to mutations of the *MECP2 *gene in Xq28 which encodes a Methyl-CpG DNA binding protein functioning as transcriptional repressor [65]. This protein is highly expressed in the CNS, in particular in the cortex, hippocampus and cerebellum. Initial psychomotor development of Rett patients is apparently normal, then regresses around 6-18 months with the appearance of stereotyped hand movements, breathing irregularities, seizures, epilepsy, loss of speech, hypotonia, ataxia and hyperreflexia. MRI studies of young Rett patients have demonstrated the presence of a mild cerebellar hypoplasia especially of lobules I-IV, whereas in adult patients a progressive cerebellar atrophy was detected and also confirmed by post-mortem neuropathologic studies of the cerebellum [66,67]. *Mecp2 *mouse mutants show cerebellar and hyppocampal-based learning deficits and develop gait incoordination [68].

#### Xq28 microduplication syndromes

Duplications of the *MECP2 *region (MIM300260), cause a syndrome of infantile hypotonia, profound intellectual deficit, autistic features and recurrent infections [69]. Neuroradiological evidence of progressive cerebellar atrophy, emerging in the second decade of life was recently reported in males of three families carrying duplications of the *MECP2 *region [70].

In 4 families with X-linked intellectual disabilities and a copy number gain of an identical 0.3 Mb region in chromosome band Xq28 including 19 annotated genes including *FLNA *and *GDI1 *(an X-linked mental retardation gene) but not *MECP2*, affected patients presented with hypotonia, severely delayed psychomotor development, mild ataxic gait, strabismus or nystagmus but no other cerebellar signs. MRI of the brain showed mildly enlarged fourth ventricle and ventricular dilatation in two unrelated patients and a classical Dandy Walker malformation with cerebellar hypoplasia and agenesis of the corpus callosum in two other affected males. Minor facial anomalies were present in all patients [71]. A fifth family with 3 affected males carrying an identical Xq28 duplication and showing mild to moderate intellectual disability associated with vermis hypoplasia was reported [72].

#### X-linked hydrocephalus

X-linked hydrocephalus with aqueductal stenosis (MIM 30700) is the most common form of hereditary hydrocephalus with an incidence of 1/30,000 in male neonates. Most cases of X-linked hydrocephalus, are caused by mutations in the *L1CAM *gene, which encodes a cellular adhesion protein implicated in the regulation of vesicle cycling through its interaction with proteins of clathrin-mediated endocytosis [73]. In patients with loss of function mutations of *L1CAM*, global vermis hypoplasia or anterior vermis hypoplasia has been observed [74].

In a few families with X-linked mental retardation and hydrocephalus mutations of *AP1S2 *(sigma subunit of the clathrin adaptor protein complex 1) were identified. Brain MRI imaging of affected males in one family showed ventricular dilatation without aqueductal stenosis, calcifications of the basal ganglia, and mild vermis hypoplasia [75,76]. Loss of function of the clathrin adaptor AP1 determines defects in synaptic vesicle cycling with accumulation of large endosome-like vacuoles, defects which are paired with reduced motor coordination and long term spatial memory in mutant mice [77].

A list of genes implicated in X-linked cerebellar dysgenesis is provided in Table 1.

**Table 1:** List of genes implicated in X-linked cerebellar dysgenesis.

SYNDROME	GENE	CEREBELLAR PHENOTYPE	CHROMOSOMAL LOCALIZATION	MIM#
**Oligophrenin-1**	***OPHN1***	cerebellar hypoplasia (vermis lobules VI-VII)	Xq12	300486

***CASK***	***CASK***	cerebellar hypoplasia	Xp11.4	300749

**Christianson**	***SLC9A6***	cerebellar atrophy	Xq26	300231

**Hoyeraal-Hreidarsson**	***DCK1***	global cerebellar hypoplasia	Xq28	300240

**X-linked sideroblastic anemia/Ataxia**	***ABC7***	global cerebellar atrophy	Xq13	300240

**Oral-facial-digital type I/X-linked Joubert**	***OFD1***	vermis hypoplasia/molar tooth sign	Xp22	311200

**Opitz GBBB**	***MID1***	anterior vermis hypoplasia	Xp22	300000

**FragileX/FXTAS**	***FMR1***	posterior vermis hypoplasia/cerebellar atrophy	Xq27	300624/300623

**Rett**	***MECP2***	cerebellar atrophy	Xq28	312750

**MECP2/Xq28 duplications**	***-***	Cerebellar atrophy/vermis hypoplasia (DWM spectrum)	Xq28	300260/300815

**X-linked heterotaxy**	***ZIC3***	vermis hypoplasia	Xq26	306955

**X-linked hydrocephalus**	***L1CAM***	global or anterior vermis hypoplasia	Xq28	307000

**Fried**	***AP1S2***	mild vermis hypoplasia	Xp22	300630

**X-linked lissencephaly with abnormal genitalia**	***ARX***	severe cerebellar hypoplasia	Xp21	300004

**X-linked Lissencephaly**	***DCX***	mild cerebellar hypoplasia (vermis)	Xq22	300067

**Oto-palato-digital type II**	***FLNA***	mild cerebellar hypoplasia	Xq28	300049

### c. X-Linked loci/syndromes with cerebellar dysgenesis

- A four generation family of Dutch descent with nine affected males presenting with severe cognitive delay, early hypotonia with progression to spasticity and contractures, choreoatetosis, seizures and a long narrow face with coarse features was reported [78]. Brain imaging studies revealed a cystic enlargement of the 4th ventricle with cerebellar hypoplasia and iron accumulation in the basal ganglia with neuroaxonal dystrophy. The disease was mapped to the long arm of the X-chromosome with a maximum lod score of 2.19 at DXS425 in Xq25-q27 [79].

- A family of Italian descent has been reported with 7 affected males, two of them showing severe congenital ataxia, generalized hypotonia, psychomotor delay, recurrent bronchopulmonary infections later developing myoclonic encephalopathy and macular degeneration [80]. Serial neuroimaging studies showed cerebellar vermis and corpus callosum hypoplasia, a cyst of the septum pellucidum and persistence of the cavum vergae. The other five affected males in the family had recurrent bronchopneumonia with severe congenital hypotonia and died within the first years of life. Immunologic investigations only showed a reduced level of IgG2 subclass in one sib. The condition was mapped to the short arm of the X-chromosome with a maximum lod score of 2.48 at DXS7099 in Xp22.33-pter [81].

- A family with a syndromic form of X-linked mental retardation characterized by absent speech, truncal ataxia, contractures and hypoplasia of the cerebellum and the brainstem revealed at neuroimaging studies was reported. Only one of the affected males developed seizures and showed changes in the basal ganglia suggesting iron deposits. The condition was mapped to Xq23-q24 [82].

- A family in which affected males showed congenital cerebellar hypoplasia, microcephaly, short stature, profound developmental delay, blindness, deafness and seizures was described. Death occurred in infancy or early childhood. This syndrome was mapped to Xq24-Xq27 [83].

- A family with 7 affected males presenting moderate to severe intellectual impairment and infantile spams was reported. Neuroimaging studies showed cerebellar atrophy or corpus callosum hypoplasia or both, in half of the affected individuals. The condition was mapped to Xp11.4-p22.11 [84].

- A family with 3 males in two generations affected with moderate intellectual deficit and prominent glabella, synophris, prognathism, generalized hirsutism and bilateral single palmar creases was described. All patients developed seizures in childhood and two of them showed a progressive gait disturbance and prolonged nerve conduction velocity and hypogammaglobulinemia. Neuroimaging studies in one patient revealed cerebellar atrophy. The disorder was mapped to Xq21.33-Xq23 [85].

Note: The clinical features of other XLCD for which no mapping data are available [86-93] have been reviewed by [5].

### Diagnosis

Family history is important to exclude autosomal or other forms of inheritance. In sporadic males or documented X-linked familiar cases, genetic testing, with the possible exception of *FMR1*, should be performed only in well selected patients with a specific clinical and neuroradiological phenotype as described in each paragraph of the present review. A precise nosological classification and a correlation between the neuroradiological phenotypes (cerebellar hypoplasia, atrophy or dysplasia, brainstem and cerebellar hypoplasia, molar tooth sign) and the genotype is still lacking but will certainly improve as the genetic causes of these conditions are being identified and also thanks to the advancement of neuroimaging techniques (eg. tractography, voxel-based morphometry). On the X-chromosome, a high incidence of causal deletions and microduplications (pathogenic copy number variations) was found, demonstrating that dysregulated expression of tightly regulated genes can disturb normal brain and cognitive development [94]. At least 1/3 of cases with *OPHN1 *or *CASK *syndrome were found to be carriers of a chromosomal or subchromosomal abnormality. It is therefore recommended, in patient with a neuroradiologically documented cerebellar disorder, especially when two or more dysmorphic features are present, to perform standard karyotype followed by array-CGH studies.

### Genetic counseling and prenatal diagnosis

Given the report of several forms of X-linked cerebellar disorders and the excess of males with ataxia [95,96], families of children with congenital cerebellar hypoplasia, atrophy or Dandy-Walker malformation with or without intellectual disability, should be counseled for high risk of X-linked inheritance. The identification of the molecular defect in couples at risk, allows early prenatal testing whereas foetal brain neuroimaging may prove uninformative.

### Management, differential diagnosis and follow-up

Optimal management of these patients requires a multidisciplinary approach with particular attention to three-generation family history and personal prenatal and post-natal history to exclude infectious, toxic or traumatic etiology. A complete physical examination focused on the presence of minor anomalies, an assessment of multiorgan involvement and the search for associated problems: neurosensorial deficits, seizures, specific learning problems is important because most of these conditions are syndromic. A cognitive and behavioural assessment (Wechsler scales, Vineland) is essential because X-linked cerebellar disorders are frequently associated with mild to severe developmental/cognitive delay. A Metabolic work-up (lactate/pyruvate ratio in blood and spinal fluid, lysosomal enzymes, isoelectrofocusing of transferrin, creatin kinase, very long chain fatty acids, plasma and urinary aminoacids and urinary organic acids chromatography) together with nerve conduction studies should be performed to exclude metabolic or neuromuscular causes (pyruvate dehydrogenase deficiency, X-linked adrenoleukodystrophy, X-linked dystrophinopathies). Neuroimaging studies of the cerebellum with particular attention to the brainstem and cerebral cortex, can be fundamental for the differential diagnosis between different X-linked cerebellar syndromes and other non cerebellar X-linked disorders that can present with ataxic symptoms (e.g Arts syndrome, characterized by ataxia, deafness and neuropathy). A clinical and neuroradiological follow-up is also necessary for the assessment of the natural history of the condition. Rehabilitation strategies should be planned for motor, cognitive and behavioral difficulties and specific manifestations such as visual impairment and seizures.

## Abbreviations

CA: Congenital ataxia; DWM: Dandy Walker malformation; XLCD: X-linked Cerebellar Dysgenesis.

## Competing interests

The authors declare that they have no competing interests.

## Authors' contributions

The present review was conceived by both authors, GZ drafted the manuscript. All authors read and approved the final manuscript.

## Acknowledgements

The author is grateful to John M Opitz (University of Utah), Eugen Boltshauser (University of Zuerich) and Jamel Chelly and his research group at the Institut Cochin (UMR8104, Université Paris Descartes) for helpful discussions and advices. This work was supported by a grant from Telethon Italy (projects GGP08145 and 492B) and the Italian Ministry of Health.
